# Lipoxins and aspirin-triggered lipoxin alleviate bone cancer pain in association with suppressing expression of spinal proinflammatory cytokines

**DOI:** 10.1186/1742-2094-9-278

**Published:** 2012-12-26

**Authors:** Shan Hu, Qi-Liang Mao-Ying, Jun Wang, Zhi-Fu Wang, Wen-Li Mi, Xiao-Wei Wang, Jian-Wei Jiang, Ya-Lin Huang, Gen-Cheng Wu, Yan-Qing Wang

**Affiliations:** 1 Department of Integrative Medicine and Neurobiology, State Key Laboratory of Medical Neurobiology, Institute of Acupuncture Research (WHO Collaborating Center for Traditional Medicine), School of Basic Medical Sciences, Shanghai Medical College, Institutes of Brain Science, Fudan University, 138 Yi Xue Yuan Road, P.O. Box 291, Shanghai 200032, China; 2 Institutes of Biomedical Sciences, Institute of Stem Cell and Regeneration Medicine, Fudan University, Shanghai, 200032, P. R. China

**Keywords:** Lipoxins, Aspirin-triggered-15-epi-lipoxinA4, Astrocytes, Neurons, Proinflammatory cytokines, Cancer-induced bone pain, Spinal cord, Rats

## Abstract

**Background:**

The neuroinflammatory responses in the spinal cord following bone cancer development have been shown to play an important role in cancer-induced bone pain (CIBP). Lipoxins (LXs), endogenous lipoxygenase-derived eicosanoids, represent a unique class of lipid mediators that possess a wide spectrum of anti-inflammatory and pro-resolving actions. In this study, we investigated the effects of intrathecal injection with lipoxin and related analogues on CIBP in rats.

**Methods:**

The CIBP model was induced by intra-tibia inoculation of Walker 256 mammary gland carcinoma cells. Mechanical thresholds were determined by measuring the paw withdrawal threshold to probing with a series of calibrated von Frey filaments. Lipoxins and analogues were administered by intrathecal (i.t.) or intravenous (i.v.) injection. The protein level of LXA4 receptor (ALX) was tested by western blot. The localization of lipoxin receptor in spinal cord was assessed by fluorescent immunohistochemistry. Real-time PCR was carried out for detecting the expression of pro-inflammatory cytokines.

**Results:**

Our results demonstrated that: 1) i.t. injection with the same dose (0.3 nmol) of lipoxin A4 (LXA4), lipoxin B4 (LXB4) or aspirin-triggered-15-epi-lipoxin A4 (ATL) could alleviate the mechanical allodynia in CIBP on day 7 after surgery. ATL showed a longer effect than the others and the effect lasted for 6 hours. ATL administered through i.v. injection could also attenuate the allodynia in cancer rats. 2) The results from western blot indicate that there is no difference in the expression of ALX among the naive, sham or cancer groups. 3) Immunohistochemistry showed that the lipoxin receptor (ALX)-like immunoreactive substance was distributed in the spinal cord, mainly co-localized with astrocytes, rarely co-localized with neurons, and never co-localized with microglia. 4) Real-time PCR analysis revealed that, compared with vehicle, i.t. injection with ATL could significantly attenuate the expression of the mRNA of proinflammatory cytokines (IL-1β and TNF-α) in the spinal cord in CIBP.

**Conclusions:**

Taken together, the results of our study suggest that LXs and analogues exert strong analgesic effects on CIBP. These analgesic effects in CIBP are associated with suppressing the expression of spinal proinflammatory cytokines.

## Background

A recent study on the prevalence of pain in cancer in 11 European countries and Israel found that 56% of patients suffered moderate to severe pain at least monthly, and 69% of patients reported pain-related difficulties with everyday activities [1,2]. Although pain has been studied in depth for decades, the pain associated with cancer is still under-treated and disruptive to the patient’s quality of life. Therefore, it is imperative to investigate the mechanism of cancer pain and to find effective therapy. However, because of the complicated mechanism of cancer pain, the underlying mechanisms are still unclear. It has been reported that cancer-induced bone pain (CIBP) is a unique pain state showing physiological characteristics of both inflammatory and neuropathic pain and changes in dorsal horn-cell phenotype [3-8]. As compared with neuropathic pain and inflammatory pain, CIBP might display physiological and pathological changes similar to those observed in the spinal cord. After the cancer cells invaded or were injected, an inflammatory response is inevitably observed, while in the later phase, the nervous system is invaded by cancer cells or other inflammatory factors and displays characteristic inflammatory responses [6,7,9,10]. Rats with CIBP display rapid expression and release of multiple inflammatory mediators, such as prostaglandin (PGE2), nerve growth factor (NGF) and proinflammatory cytokines including interleukin-1β (IL-1β), interleukin-6 (IL-6) and tumor necrosis factor-α (TNF-α), at the spinal cord. These mediators participate in the pathogenesis of CIBP [10-12]. Recently, accumulating evidence supports an important role of spinal non-neuronal cells, such as astrocytes and microglia, in the regulation of nociception [12,13]. Certainly, neuroinflammation is a significant characteristic of the entire pathological process of CIBP.

Accumulating evidence shows that most inflammatory processes are self-limiting and self-resolving systems, which are known as an active endogenous process aimed at protecting the host from exacerbated inflammation [14-16]. The outcome depends on the balance between pro-inflammatory mediators and anti-inflammatory mediators *in vivo*[17]. Lipoxins (LXs) belong to a class of eicosanoid that is generated from arachidonic acid via the sequential actions of lipoxygenases and subsequent reactions to yield specific trihydroxytetraene-containing eicosanoids [18]. As the first recognized class of anti-inflammatory lipid-based autacoids, LXs mediate a number of processes, including the regression of pro-inflammatory cytokine production, inhibition of cell proliferation, promotion of the recruitment of monocytes and stimulation of non-phlogistic phagocytosis of apoptotic leukocytes by monocyte-derived macrophages, suggesting that lipoxins may act as endogenous ‘braking signals’ in host defense, inflammation and hypersensitivity reactions [17,19,20]. LXA4 and LXB4 are positional isomers that possess potent cellular and *in vivo* actions [21]. Moreover, aspirin has a direct impact on the LX circuit by triggering the biosynthesis of endogenous epimers of LXs, termed aspirin-triggered-15-epi-lipoxin A4 (ATL), which share the potent anti-inflammatory actions of LXs [15,21,22].

It has been reported that i.t. injection of LXs can attenuate inflammatory pain and neuropathic pain [23,24]. Due to the sustained and robust spinal neuroinflammation that characterizes CIBP, we hypothesized that i.t. injection with LXs may be a novel strategy that mimics the action of endogenous anti-inflammatory and pro-resolution lipid mediators to alleviate CIBP. Therefore, the present study was designed to explore the possible analgesic effect of LXs on the rat model of CIBP.

## Methods

### Animals

Experiments were performed on pathogen-free adult female Sprague–Dawley (SD) rats (Shanghai Laboratory Animal Center, Chinese Academy of Sciences, Shanghai, China) weighing 160 g to 180 g. Animals were housed in groups of 4 to 6 per cage and maintained on a 12:12 hour light–dark cycle and constant room conditions (temperature, 22 ± 2°C; humidity, 55% ± 10%) with free access to food and water. Prior to experimental manipulation, rats were habituated in the animal room for at least one week after delivery. All experimental protocols and animal-handling procedures were performed according to protocols approved by the Animal Care and Use Committee (ACUC) of Fudan University and were consistent with the National Institutes of Health Guide for the Care and Use of Laboratory Animals and the International Association for the Study of Pain’s (IASP) guidelines for pain research [24]. All efforts were made to minimize the number of animals used and to minimize their suffering.

### Preparation of cells

Walker 256 rat mammary gland carcinoma cells (generously provided by the Institute of Radiation Medicine, Fudan University) were injected into the abdominal cavities of female SD rats weighing 60 g to 80 g (2 × 10^7^ cells/0.5 ml). After 6 to 7 days, cancerous ascites was harvested in a sterile fashion, and the carcinoma cells were subsequently washed with PBS, pH=7.2, three times by centrifugation for 3 minutes at 1200 rpm. The pellet was resuspended with PBS and adjusted to an appropriate concentration (1 × 10^8^/ml). The cell suspension was maintained on ice until injection.

### Surgical procedure

As previously described [5,25], rats (except the naive group) were anesthetized with chloral hydrate (400 mg/kg, i.p.). Superficial incisions were made in the skin overlying the patella after disinfection with 75% v/v ethanol in order to expose the tibia head with minimal damage. After Walker 256 carcinoma cells were prepared, 4 μl of cells followed by 4 μl PBS were slowly injected into the right tibia cavity of each rat using a 10-μl microinjection syringe. The syringe was left in place for an additional two minutes to prevent the carcinoma cells from leaking out along the injection track. The injection site was closed using bone wax as the syringe was removed to prevent tumor cell overflow. The sham group rats were treated in the same way and injected with 8 μl of PBS instead of tumor cells. All rats were given gentamycin (40 mg/kg, i.p.) for three consecutive days to avoid wound infection.

### Drug administration

To determine the effects of LXs on the bone cancer-induced mechanical allodynia, a single dose of LXA4 (Merck, Darmstadt, Germany), ATL (Merck, Darmstadt, Germany), LXB4 (Cayman, Ann Arbor, MI, USA) or normal saline (NS) was administered in a volume of 20 μl to rats via lumbar puncture, as previously reported [26]. Briefly, the rats were anesthetized with isoflurane. The lumbar region was disinfected with 75% v/v ethanol after hair shaving, and the intervertebral spaces were widened by placing the animal on a plexiglass tube. Next, a 29-gauge microinjection syringe needle filled with 20 μl of drug was inserted via the L5-6 interspace. The correct subarachnoid positioning of the tip of the needle was verified by a tail- or paw-flick response immediately after inserting the needle.

### Behavioral test

The paw withdrawal threshold (PWT) was measured using von Frey hairs (Stoelting, Wood Dale, Illinois, USA) Each rat was placed individually into a plexiglass chamber containing 1.5-mm diameter holes in a 5-mm grid of perpendicular rows throughout the entire area of the metal platform [27]. After acclimation to the test chamber, a series of eight calibrated von Frey hairs (0.4, 0.6, 1.4, 2, 4, 6, 8 and 15 g) were applied to the central region of the plantar surface of one hind paw. The tester was blinded with respect to group. Each von Frey hair was held for approximately 1 to 2 seconds with a 5-minute interval between applications. A trial began with the application of 2.0 g von Frey hair. A positive response was defined as a brisk withdrawal of the hind paw upon stimulation. When a positive response to a given hair occurred, the next lower von Frey hair was applied, and when a negative response occurred, the next higher hair was applied. The tests consisted of five more stimuli after the first change in response occurred, and the PWT was converted to the tactile response threshold using an adaptation of the Dixon up-down paradigm, as described previously [28]. All the tests were performed in a blinded fashion with respect to the drugs injected.

### Western blot

The L4-L5 segments of the spinal cord were homogenized and subjected to SDS-PAGE. Membranes were incubated with antibodies against ALX (1:4000, United States Biological, Swampscott, Massachusetts, USA). The signal was detected with chemiluminescent reagents (ECL kit, Pierce, Rockford, Illinois, USA). The membranes were re-blotted with GAPDH (1:10000, KangCheng, Shanghai, China). The intensity of immunoreactive bands was quantified using ImageQuant software (Molecular Dynamics).

### Immunohistochemistry

On day 11 after surgery, rats were deeply anesthetized with chloral hydrate (400 mg/kg, i.p.) and perfused intracardially with saline followed by 4% paraformaldehyde in 0.1 M phosphate buffer (PB, pH 7.4). The L4-L5 segments of the spinal cord were subsequently removed, post-fixed in the same fixative for 4 hours at 4°C, and immersed in a 10% to 30% sucrose solution in PB gradient for 24 to 48 hours at 4°C for cryoprotection. Transverse spinal sections (free-floating, 30 μm) were cut in a freezing microtome (Leica CM1900, Munich,Germany) and processed for immunofluorescence. All of the sections were blocked with 10% normal goat serum in 0.01 M PBS (pH 7.4) with 0.3% Triton-X-100 for 1 hour at 37°C and incubated overnight at 4°C with rabbit anti-ALX (1:400, United States Biological, Swampscott, Massachusetts, USA) primary antibody in PBS with 1% normal goat serum and 0.3% Triton X-100. Following three 15-minute rinses in 0.01 M PBS, the sections were incubated in Alexa Fluor 594-conjugated secondary antibody (red, 1:1000, Invitrogen, Carlsbad, California, USA) for 1 hour at 37°C and were washed in PBS. Omission of the primary antibody served as a negative control. The specificity of the anti-ALX antibody was also tested by Western Blot. For double immunofluorescence, sections were incubated with a mixture of rabbit anti-ALX and mouse anti-NeuN (1:500, Millipore, Billerica, Marriott, USA), mouse anti-CD11b (1:200, Chemicon, Billerica, Marriott, USA), mouse anti-GFAP (1:1000, Thermo, Waltham, Massachusets, USA) antibodies respectively at 37°C for 1 hour and at 4°C overnight. Next, the sections were incubated with a mixture of goat Alexa Fluor™ 594-conjugated (red, 1:1000, Invitrogen) and goat Alexa Fluor™ 488-conjugated (green, 1:1000, Invitrogen) secondary antibodies for 1 hour at 37°C. All sections were coverslipped with a mixture of 80% glycerin in 0.01 M PBS, and images were captured using a multiphoton laser point scanning confocal microscopy system (Olympus Fluoview FV1000, Leica TCS SP5, Germany).

All of the testing (including behavior, western blot, immunohistochemistry and real-time PCR) was conducted by experimenters who were blind to the experimental conditions.

### Real-time quantitative PCR

Total RNA was isolated from L4-L6 spinal cord using TRIzol reagent (Invitrogen) according to the manufacturer’s instructions. Quantification of mRNA levels of IL-1β, IL-6, TNF-α and GAPDH were analyzed by SYBR Green qRT-PCR detection (iCycler iQ® real-time PCR detection system, Bio-Rad, Hercules City, California, USA), with each sample being run in duplicate. Samples of cDNA from naive, cancer with NS and cancer with ATL animals 2 hours after drug injection were analyzed simultaneously by real-time PCR. The PCR mixture was prepared by using the multiplex real-time PCR protocol according to the manufacturer’s instructions. A total of 2 μl of reverse transcription product from each sample was used as the template in a 25-μl reaction mixture. The size and sequence of each primer and the number of cycles used are given in Table 1[29]. The standard curve of each primer showed that the amplification efficiency was 90% to 100% (data not shown). Upon completion of the PCR, the amount of target message in each sample was estimated based on the threshold cycle number (Ct). Average Ct values were normalized to average Ct values for GAPDH mRNA from the same cDNA preparations. These values were entered into the equation 2^−ΔΔCT^ to solve for the relative exponential PCR amplification of each gene for each animal [30,31]. The results presented in this study are expressed as fold increases over control values.

**Table 1:** Seqences of the forward and reverse primers and PCR conditions used for RT-PCR

**Genbank accession numbers**	**Target gene**	**Primers**	**PCR conditions (temperature/time)**		
			**Denature**	**Anneal**	**Extend**
NM_017008	GAPDH	Foward: 5^′^cccttcattgacctcaactac-3^′^	94°C/45 seconds	60°C/1 minute	72°C/1 minute
		Reverse: 5^′^-cttctccatggtggtgaagac-3^′^			
NM_031512	IL-1β	Forward: 5^′^-atgagagcatccagcttcaaatc-3^′^	94°C/45 seconds	58°C/1 minute	72°C/1 minute
		Reverse: 5^′^-cacactagcaggtcgtcatcatc-^′^3			
NM_012589	IL-6	Forward: 5^′^-gacaaagccagagtccttca-3^′^	94°C/45 seconds	58°C/1 minute	72°C/1 minute
		Reverse: 5^′^-actaggtttgccgagtagac-3^′^			
X66539	TNF-α	Forward: 5^′^-cgagatgtggaactggcaga-3^′^	94°C/45 seconds	58°C/1 minute	72°C/1 minute
		Reverse: 5^′^-ctacgggcttgtcactcga-3^′^			

### Statistical analysis

All data are presented as mean ± standard error of the mean (SEM). The statistical significance of differences between groups was analyzed with Student’s T-test or one-way analysis of variance (ANOVA) following the least-significant difference (LSD) post-test or Bonferroni post-test. *P*<0.05 was set as the threshold of significance.

## Results

### The development of CIBP

**Figure 1:**
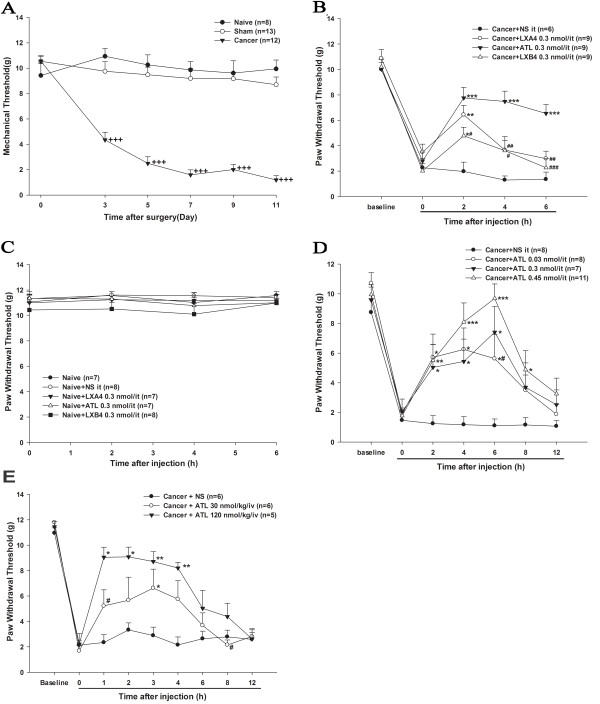
**Lipoxins and analogues alleviate mechanical allodynia in the CIBP model induced by intra-tibia injection of Walker 256 carcinoma cells (4×10**^**5**^**).** (**A**) The time course of the development of mechanical allodynia after cancer cell inoculation. ^**+++**^*P*<0.001 versus the sham group (by Bonferroni test). (**B**) Comparisons were also made between the effects of the ATL, LXA4 and LXB4 treatments on rats with CIBP on day 7 after surgery. Data are expressed as means ± SEM. *P<0.05, **P<0.01, ***P<0.001 versus cancer + NS (by Bonferroni test). ^**##**^*P*<0.01, ^**###**^*P*<0.001 versus cancer +ATL (by T-test). (**C**) Effects of i.t. LXA4, LXB4 and ATL on the mechanical allodynia of naive rats (by Bonferroni test). (**D**) Anti-allodynia effects of i.t.ATL at different doses on rats with CIBP. On day 11 after surgery, the cancer groups were given different doses of ATL by i.t. injection. Note that the dose of 0.45 nmol showed a better and longer analgesic effect from 2 to 8 hours than the other doses. *P<0.05, **P<0.01, ***P<0.001 versus cancer + NS (by LSD test). ^**#**^*P*<0.05 versuscancer + ATL 0.45 nmol (by T-test). (**E**) Anti-allodynia effects of i.v. ATL at different doses on rats with CIBP. On day 11 after surgery, the three groups were injected i.v. with NS or different doses of ATL (10 nmol/kg or 120 nmol/kg). ^**#**^*P*<0.05 versus cancer +ATL 30 nmol/kg (by T-test). Data are expressed as means ± SEM. *P<0.05, **P<0.01, ***P<0.001 versus cancer + NS (by Bonferroni test).ATL, aspirin-triggered-15-epi-lipoxin A4; CIBP, cancer-induced bone pain; i.t., intrathecal; i.v., intravenous; LSD, least-significant difference; LX, lipoxin; NS, normal saline; SEM, standard error of the mean.

After the baseline behavioral test, the rats in the cancer group received carcinoma cells (Walker 256 carcinoma cells, 4×10^5^) that were injected into the right tibia cavity, as previously described (day 0). The sham group received an equal volume of PBS (day 0). The naive group received no treatment. In order to assess the development of mechanical allodynia in CIBP, PWT was tested on day 3, 7, 9 and 11after surgery. On day 3 after surgery, the rats in the cancer group displayed a profound decrease in PWT to von Frey hair stimulation in the ipsilateral right limb (*P*<0.001, Bonferroni test) compared with the sham group. This decrease lasted until day 11 when the observation ended. In contrast, there was no significant difference in PWT between the naive group and the sham group at the different time points (*P*>0.05, Bonferroni test) (Figure 1A).

### Intrathecal injection with LXA4, ATL and LXB4 reduces the mechanical allodynia in CIBP

In order to examine the analgesic effects of LXs on the mechanical allodynia in CIBP, on day 7 after surgery, cancer rats were randomly divided into four groups: one group was treated i.t. with NS (20 μl) as vehicle, and the others were treated i.t. with equal doses of LXA4, LXB4 or ATL (0.3 nmol/20 μl). The ipsilateral PWT was tested at 2, 4 and 6 hours after drug administration. Compared with vehicle-treated animals, the PWT was profoundly increased from 2 to 6 hours in the ATL-injected animals (*P*<0.001, Bonferroni test), while this measure was significantly increased after 2 hours in the LXA4-injected animals (*P*<0.01, Bonferroni test) and in the LXB4-injected animals (*P*<0.05, Bonferroni test) (Figure 1B). However, i.t. treatment with equimolar doses of LXA4, LXB4 or ATL did not alter the nociceptive thresholds of the naive animals (*P*>0.05) (Figure 1C, Bonferroni test).

### Intrathecal injection with different doses of ATL alleviates mechanical allodynia in CIBP

We assessed the effects of i.t. with different doses of ATL on the ipsilateral PWT of animals on day 11 after surgery. Cancer rats were randomly divided into four groups: one group was treated with i.t. NS (20 μl) as vehicle, and the others were treated i.t. with different doses of ATL (0.03, 0.3 or 0.45 nmol/20 μl). PWT was tested at 2, 4, 6, 8 and 12 hours after drug administration. Compared with the vehicle-treated animals, the animals treated i.t. with ATL (0.03, 0.3 or 0.45 nmol) showed significant increases in PWT from 2 to 6 hours (*P*<0.05, LSD test). The dose of 0.45 nmol showed longer effects (for 8 hours) (Figure 1.D, LSD test). These results indicate that a single administration of ATL could reduce the mechanical allodynia in CIBP.

### Intravenous ATL attenuates mechanical allodynia in CIBP

To assess the possible analgesic effect of systemically administered ATL, the effects of i.v. ATL on the mechanical allodynia in cancer rats were observed on day 11 after surgery. From 1 to 4 hours after the injection of 30 or 120 nmol/kg ATL, the PWT increased significantly compared with vehicle (Figure 1E, Bonferroni test) indicating an anti-allodynic effect of i.v. ATL in cancer rats.

### Expression of ALX in the spinal cord

**Figure 2:**
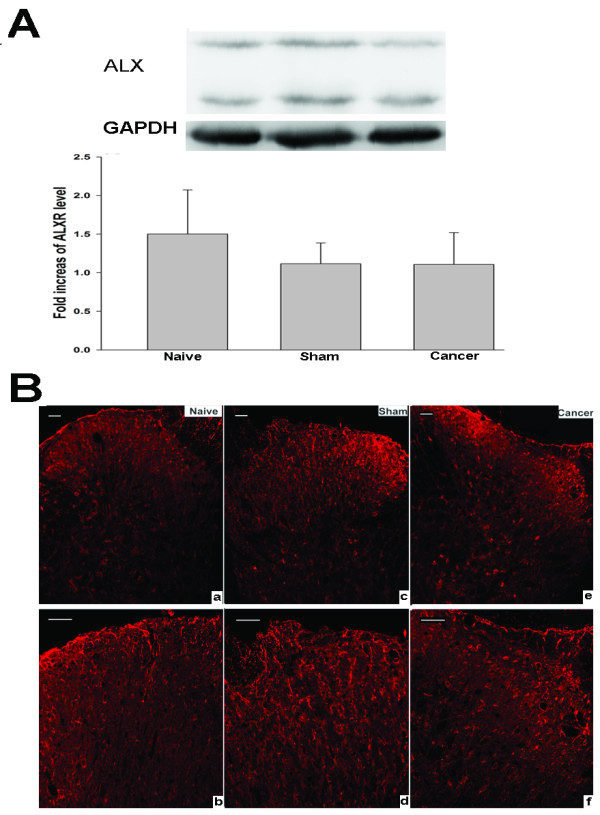
**Localization and expression level of ALX among the naive, sham and cancer groups on day 11 after surgery.** (**A**) Western blot detection of ALX expression in the spinal cord of rats. Data are expressed as means ± SEM (n=4) (by Bonferroni test). (**B**) Photomicrographs showing the expression of ALX in the dorsal horn of the ipsilateral spinal cord of rats. Immunohistological staining was carried out on the spinal cord sections from the naive (**a-b**), sham (**c-d**) and cancer (**e-f**) groups. Bar 100 μm. ALX, lipoxin A4 receptor; SEM, standard error of the mean.

**Figure 3:**
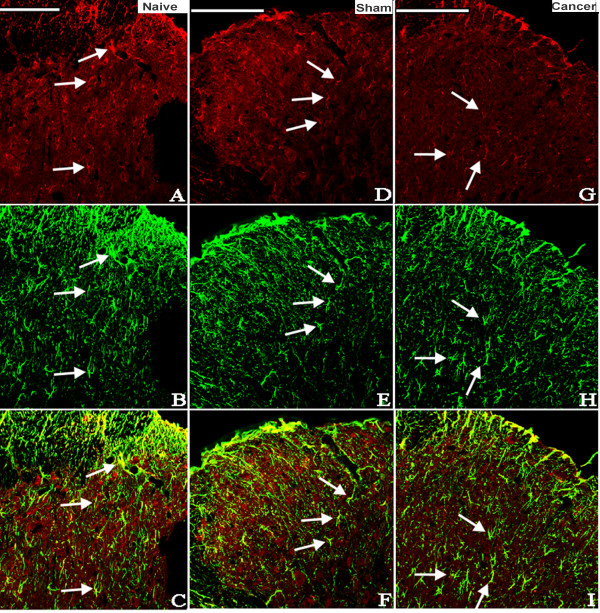
**Photomicrographs showing the expression of GFAP (astrocyte marker) (green) and ALX (red) on the dorsal horn of the ipsilateral spinal cord of rats.** Examples of double labeling are indicated with white arrows. Immunohistological processing of spinal cord sections from the naive (**A-C**), sham (**D-F**) and cancer (**G-I**) groups showed obvious co-localization of ALX-like immnoreactivity and the astrocyte marker GFAP (n=4). Bar 100 μm. ALX, lipoxin A4 receptor; GFAP, glial fibrillary acidic protein.

**Figure 4:**
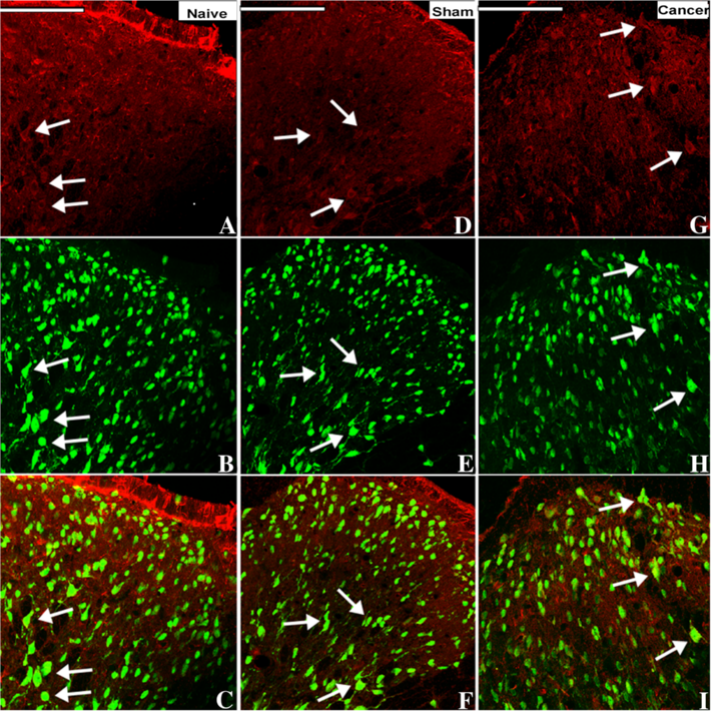
**Photomicrographs showing the expression of NeuN (neuron marker) (green) and ALX (red) on the dorsal horn of the ipsilateral spinal cord of rats.** Examples of double labeling are indicated with white arrows. Immunohistological processing of spinal cord sections from the naive (**A-C**), sham (**D-F**) and cancer (**G-I**) groups showed that ALX-like immunoreactivity is partly co-localized with the neuronal marker NeuN (n=4). Bar 100 μm. ALX, lipoxin A4 receptor.

**Figure 5:**
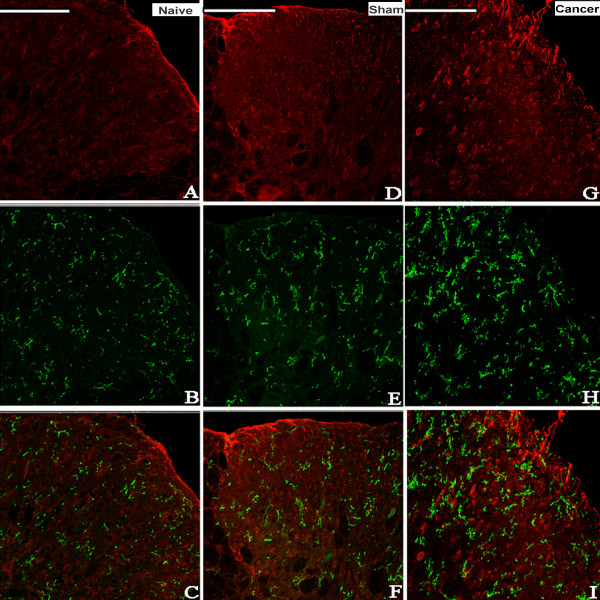
**Photomicrographs showing the expression of CD11B (microglial marker) (green) and ALX (red) on the dorsal horn of the ipsilateral spinal cord of rats.** Immunohistological processing of spinal cord sections from the naive (**A-C**), sham (**D-F**) and cancer (**G-I**) groups showed no co-localization of ALX-like immunoreactivity and the microglial marker CD11B (n=4). Bar 100 μm. ALX, lipoxin A4 receptor.

The possible distribution of ALX in the spinal cord was detected by immunohistochemistry. Lumbar spinal cords from the naive, sham and cancer groups were sectioned and incubated with ALX antibody. Similar and moderate ALX-like immunoreactivity was observed in the spinal cord of naive, sham and cancer rats (Figure 2B). No significant difference in the level of the expression of the ALX protein was found among naive, sham or cancer groups (Figure 2A). Double labeling of spinal sections revealed that the ALX-like immunoreactivity was mainly co-localized with the astrocyte marker glial fibrillary acidic protein (GFAP; Figure 3), partly co-localized with the neuronal marker NeuN (Figure 4) but not with the microglia marker CD11B (Figure 5).

### Effects of ATL on the expression of the mRNA of pro-inflammatory cytokines in CIBP

**Figure 6:**
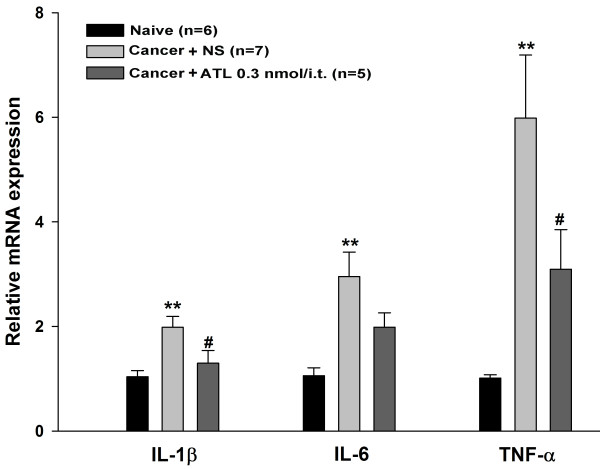
**Effects of intrathecal ATL on the expression of IL-1β, IL-6 and TNF-α mRNA assessed by RT-PCR in the spinal cords of rats with CIBP.** The L4-L5 spinal cords were used to test 2 hours after i.t. injection with ATL (0.3 nmol/20 μl) or NS (20 μl) on day 11 after surgery. Data are expressed as means ± SEM. **P<0.01 versus naive, #*P*<0.05 versus cancer + NS (by LSD test), ATL, aspirin-triggered-15-epi-lipoxin A4; CIBP, cancer-induced bone pain; i.t., intrathecal; LSD, least-significant difference; NS, normal saline.

To investigate the molecular mechanisms associated with the anti-allodynic effect of ATL in rats with CIBP, we evaluated the expression of the mRNA of several pro-inflammatory mediators, including IL-1β, IL-6 and TNF-α in the spinal cord by real-time PCR. Rats were randomly divided into three groups: naive, cancer with NS and cancer with ATL. As compared with the naive group, cancer with saline groups showed significant increases in pro-inflammatory mediators (*P*<0.01, LSD test). I.t. injection of ATL to the rats with CIBP significantly decreased the expression of the mRNA of IL-1β and TNF-α (*P*<0.05, LSD test) (Figure 6).

## Discussion

The present study demonstrated that i.t. treatment with LXA4, LXB4 or ATL could significantly alleviate the mechanical allodynia in CIBP. ATL showed the longest and most potent analgesic effect compared to LXA4 and LXB4. Further analysis showed that the increased expression of IL-1β and TNF-α in CIBP were significantly inhibited after i.t. injection with ATL. Immunohistochemistry revealed that ALX was mainly co-localized with the astrocytes and partly co-localized with the neurons but not with microglia. The present study suggests that ATL might exert its anti-neuroinflammation effects in CIBP through the ALX receptor expressed on astrocytes and/or some neurons at the spinal cord.

Many reports suggest that the ALX was identified and cloned in various cell types, including polymorphonuclear cells, monocytes, activated T cells, intestinal enterocytes and synovial fibroblasts [14,22]. In our study, the analgesic effects observed after i.t. injection with LXA4, LXB4 and ATL indicated that this class of lipid mediators acts on specific targets in the spinal cord. Recently, evidence from two independent groups revealed the existence of ALX expression in rat primary astrocytes [24,32] and microglia [33] at both the mRNA and protein levels. However, the present results showed that the ALX was mainly co-localized with astrocytes, sometimes co-localized with neurons, and did not co-localize with microglia, which is inconsistent with the previous reports. These discrepancies may be due to the different models and contexts (*in vivo* or *in vitro*). It has been demonstrated that non-neuronal cells may play an important role in the spinal facilitation of pain processing [34,35], and LXs may act through ALX distributed on astrocytes and neurons to participate in the development and maintenance of chronic pain. This point needs to be elucidated in further investigations.

The significantly superior analgesic effect of ATL compared to the other drugs may have stemmed from the trihydroxytetraene structure of native lipoxins, which is sensitive to metabolic inactivation by dehydrogenation, but ATL is more resistant to metabolic inactivation than is the native LXs [21,22]. It has been reported that i.v. treatment with LXA4, LXB4 or ATL significantly alleviated the heat hyperalgesia in a carrageenan-induced inflammatory pain model [24]. The present study revealed similar effects of LXs and analogues on CIBP.

Furthermore, repeated i.t. injection of ATL had a therapeutic analgesic effect on neuropathic pain in a chronic compression of dorsal root ganglia (CCD) model [23]. Since the multiple effects of LXs and analogues include anti-inflammatory and anti-cancer effects [36-38], the possible therapeutic effect of chronic systemic administration of LXs and analogues needs to be assessed soon.

Early reports demonstrated that LXs play an important role in pain processing by regulating communication between the immune and sensory nervous systems [39], which has been supported by research regarding the analgesic effects of LXs on inflammation pain and neuropathic pain [23,24,40]. It has been reported that LXA4 and ATL could interfere with the mitogen-activated protein kinase (MAPK) signaling pathway, inhibit the activation of NF-kappa B and AP-1, and consequently control the expression of pro-inflammatory cytokines [21,22,41]. Therefore, ATL may alleviate mechanical allodynia in CIBP by inhibiting the MAPK signaling pathway and NF-kappa B activation to inhibit the production of pro-inflammatory mediators. However, our current *in vivo* study revealed little effect of ATL on the spinal MPAK signaling pathways (data not shown). These warrant further study and *invitro* studies are underway by culturing spinal neurons and glial cells, respectively.

Interestingly, it has been reported that an LX analogue can elevate the mRNA of both suppressors of cytokine signaling–1 (SOCS-1) and SOCS-2, two of the endogenous inhibitors of cytokine-elicited signaling pathways, in the kidney in ischemic acute renal failure in mice [42]. Our preliminary experiments showed that ATL-treated rats also displayed increased spinal mRNA levels for SOCS-1 (data not shown). Since the putative role for SOCSs as endogenous inhibitors of cytokine bioactivities transduced through JAK-STAT signal transduction pathways [43,44], the finding of decreased mRNA levels for IL-1β, IL-6 and TNF-α in association with increased expression of SOCS-1 suggests a possible mechanism through which lipoxins could modulate cytokine bioactivity and, hence, attenuate spinal neuroinflammation conditions in rats with CIBP. The LXs may exert their analgesic effect through the ALX on astrocytes and neurons via its multipronged effects on the neuroinflammation milieu as well as neural activity in the spinal cord. However, our *in vivo* study revealed little effect of ATL on these signal pathways (data not shown). These warrant further study and *in vitro* studies are being conducted by culturing spinal neurons and glial cells, respectively.

Taken together, the results of the present study demonstrated for the first time that i.t. injection with LXs could strongly attenuate the mechanical allodynia in CIBP. The increased expression of pro-inflammatory mediators in CIBP was significantly attenuated by i.t.ATL. This study indicates that LXs and analogues could alleviate CIBP with sustained efficacy and these findings point to novel therapeutic targets for analgesia in CIBP.

## Abbreviations

ALX: Lipoxin A4 receptor; ATL: Aspirin-triggered-15-epi-lipoxin A4; CIBP: Cancer-induced bone pain; Ct: Cycle threshold; GFAP: Glial fibrillary acidic protein; IL: Interleukin; i.t.: Intrathecal; i.v.: Intravenous; LSD: Least significant difference; LXs: Lipoxins; MAPK: Mitogen-activated protein kinase; NGF: Nerve growth factor; NS: Normal saline; PB: Phosphate buffer; PBS: Phosphate-buffered saline; PGE2: Prostaglandin; PWT: Paw withdrawal threshold; qRT-PCR: Quantitative reverse transcriptase-polymerase chain reaction; SD: Sprague–Dawley; SEM: Standard error of the mean; SOCS: Suppressors of cytokine signaling; TNF-α: Tumor necrosis factor α.

## Competing interests

The authors declare that they have no competing interests.

## Authors’ contributions

SH carried out the major part of the study. SH, QLMY, ZFW and XWW performed the animal surgery and the behavioral tests. SH and JW carried out the Western blot and Real-Time PCR study. SH drafted the manuscript. JWJ, WLM and YLH carried out part of the immunofluorescence study. GCW revised the manuscript. YQW conceived and designed the study. All authors read and approved the final manuscript.
